# M_1_ muscarinic receptor activation reduces the molecular pathology and slows the progression of prion-mediated neurodegenerative disease

**DOI:** 10.1126/scisignal.abm3720

**Published:** 2022-11-15

**Authors:** Louis Dwomoh, Mario Rossi, Miriam Scarpa, Elham Khajehali, Colin Molloy, Pawel Herzyk, Shailesh N. Mistry, Andrew R. Bottrill, Patrick M. Sexton, Arthur Christopoulos, P. Jeffrey Conn, Craig W. Lindsley, Sophie J. Bradley, Andrew B. Tobin

**Affiliations:** 1 Centre for Translational Pharmacology, Institute of Molecular, Cell and Systems Biology, College of Medical, Veterinary and Life Sciences, University of Glasgow, Glasgow G12 8QQ, UK; 2 Drug Discovery Biology, Monash Institute of Pharmaceutical Sciences, Monash University, Parkville, VIC 3052, Australia; 3 Department of Anatomy and Physiology, School of Biomedical Sciences, Faculty of Medicine, Dentistry and Health Sciences, University of Melbourne, Parkville, VIC 3010, Australia; 4 School of Pharmacy, University of Nottingham, Nottingham NG7 2RD, UK; 5 Research Technology Platforms, University of Warwick, School of Life Sciences, Coventry CV4 7AL, UK; 6 Australian Research Council Centre for Cryo-electron Microscopy of Membrane Proteins, Monash Institute of Pharmaceutical Sciences, Monash University, Parkville, VIC 3052, Australia; 7 Warren Centre for Neuroscience Drug Discovery, Vanderbilt University, Nashville, TN 37232, USA

## Abstract

Many dementias are propagated through the spread of “prion-like” misfolded proteins. This includes prion diseases themselves (such as Creutzfeldt-Jakob disease) and Alzheimer’s disease (AD), for which no treatments are available to slow or stop progression. The M_1_ acetylcholine muscarinic receptor (M_1_ receptor) is abundant in the brain, and its activity promotes cognitive function in preclinical models and in patients with AD. Here, we investigated whether activation of the M_1_ receptor might slow the progression of neurodegeneration associated with prion-like misfolded protein in a mouse model of prion disease. Proteomic and transcriptomic analysis of the hippocampus revealed that this model had a molecular profile that was similar to that of human neurodegenerative diseases, including AD. Chronic enhancement of the activity of the M_1_ receptor with the positive allosteric modulator (PAM) VU0486846 reduced the abundance of prion-induced molecular markers of neuroinflammation and mitochondrial dysregulation in the hippocampus and normalized the abundance of those associated with neurotransmission, including synaptic and postsynaptic signaling components. PAM treatment of prion-infected mice prolonged survival and maintained cognitive function. Thus, allosteric activation of M_1_ receptors may reduce the severity of neurodegenerative diseases caused by the prion-like propagation of misfolded protein.

## Introduction

Many neurodegenerative diseases are associated with the abnormal aggregation and deposition of specific cellular proteins (1, 2). In prion diseases, which include Kuru and Creutzfeldt-Jakob disease in humans, scrapie in sheep, bovine spongiform encephalopathy in cows, and murine prion disease in mice, normal cellular prion protein (PrP^C^) misfolds scrapie prion protein (PrP^Sc^) and forms filamentous aggregates. The seeding of aggregated PrP^Sc^ acts to nucleate the assembly of larger filamentous aggregates through a process of self-propagation wherein normal PrP^C^ is recruited to aggregates, causing further misfolding and propagation of PrP^Sc^ filaments (3). In this way, PrP^Sc^ spreads in a manner akin to an infection (4). This process appears to be a common feature of most neurodegenerative diseases where specific cellular proteins—huntingtin protein in Huntington’s disease, α-synuclein in Parkinson’s disease, and β-amyloid (Aβ) and hyperphosphorylated tau in Alzheimer’s disease (AD)—misfold, nucleate, and spread in an infectious manner that is described as “prion-like” (2, 4, 5). Despite an appreciation of this process and considerable effort by both academia and industry, attempts to halt or slow this process—particularly in AD—have been unsuccessful (6–8).

Although not able to change the course of disease, the current frontline treatment for AD targets symptomatic memory impairment associated with the loss of acetylcholine cholinergic neurons that originate from the basal forebrain nuclei and innervate limbic and neocortical structures (9, 10). By the pharmacological inhibition of cholinesterases (the enzymes responsible for acetylcholine catabolism), acetylcholine levels in patients with AD are increased, thereby counteracting the deficit in cholinergic transmission (11). Whereas this approach has some limited clinical benefit in the symptomatic treatment of memory loss in early stages of disease (12–14), there is no consistent evidence that this approach can slow the progression of disease. Despite this, there are emerging reports that activation of postsynaptic acetylcholine receptors of the acetylcholine muscarinic receptor family, which consist of five receptor subtypes (M_1_ to M_5_ receptors), but particularly the M_1_ receptor subtype, can offer neuroprotection in the context of neuro-degenerative disease (15, 16). This is particularly exciting given that activation of the M_1_ receptor is widely considered as a promising strategy for the treatment of memory loss in AD due to the high expression of this receptor subtype in the cortex and hippocampus (15, 17) and robust procognitive effects after receptor activation in preclinical animal models (15, 18–20). Combined, these studies suggest that selective targeting of the M_1_ receptor might have a dual benefit in AD through restoration of cognitive function and neuroprotection that would slow disease progression.

The barrier to testing this hypothesis in the clinic is the development of drugs that selectively activate the M_1_ receptor, because the orthosteric acetylcholine binding site is nearly identical between the five muscarinic receptor subtypes (21). Hence, generation of subtype-selective orthosteric agonists is very challenging (22). An alternative approach that we and others have adopted is to target an allosteric binding pocket on the extracellular surface of the receptor (21). Agents that bind to this site can act by increasing the sensitivity of the receptor to acetylcholine (23). These positive allosteric modulators (PAMs) have the advantage of being highly selective for the M_1_ receptor while maintaining the spatiotemporal profile of cholinergic signaling because they act cooperatively with the natural ligand acetylcholine. These features are considered to be the primary reasons for PAMs showing reduced adverse responses normally associated with prolonged activation by orthosteric M_1_ receptor agonists (24, 25).

Our initial studies investigated the activity of the prototypical M_1_ receptor PAM, benzyl quinolone carboxylic acid (BQCA) (20), in murine prion disease. This is a terminal neurodegenerative disease where there is a progressive loss of hippocampal neurons (26), including a disruption of hippocampal cholinergic innervation with associated deficits in learning and memory, which, we have shown, can be restored by treatment with clinical cholinesterase inhibitors (15). In this model, we found that a single dose of BQCA before training in a fear conditioning protocol restored defective learning and memory in murine prion disease, whereas chronic daily treatment for several weeks slowed disease progression (15).

Here, using global proteomic and transcriptomic analysis of a murine model of prion disease, we found that prion accumulation and aggregation induce adaptive responses, including neuroinflammation and up-regulation of protein markers, as well as indicators of synaptic loss and mitochondrial dysfunction, and that these profiles were ameliorated and even normalized by the next-generation M_1_ PAM, VU0486846 (VU846) (27, 28). VU846 also improved the behavioral symptoms and extended the survival of prion-diseased mice. The molecular profiles of mouse prion disease overlapped with those associated with human neurodegenerative disease, particularly AD, in which the loss of M_1_ receptor signaling may contribute to disease pathology. We conclude that M_1_ PAMs exhibit therapeutic potential for slowing the progression of neurodegenerative disease in mice by regulating adaptive, neuroinflammatory responses that are common features of the molecular pathology of brain diseases caused by the propagation of misfolded protein.

## Results

### M_1_ PAMs restore learning and memory and prolong survival in murine prion disease

**Fig. 1:**
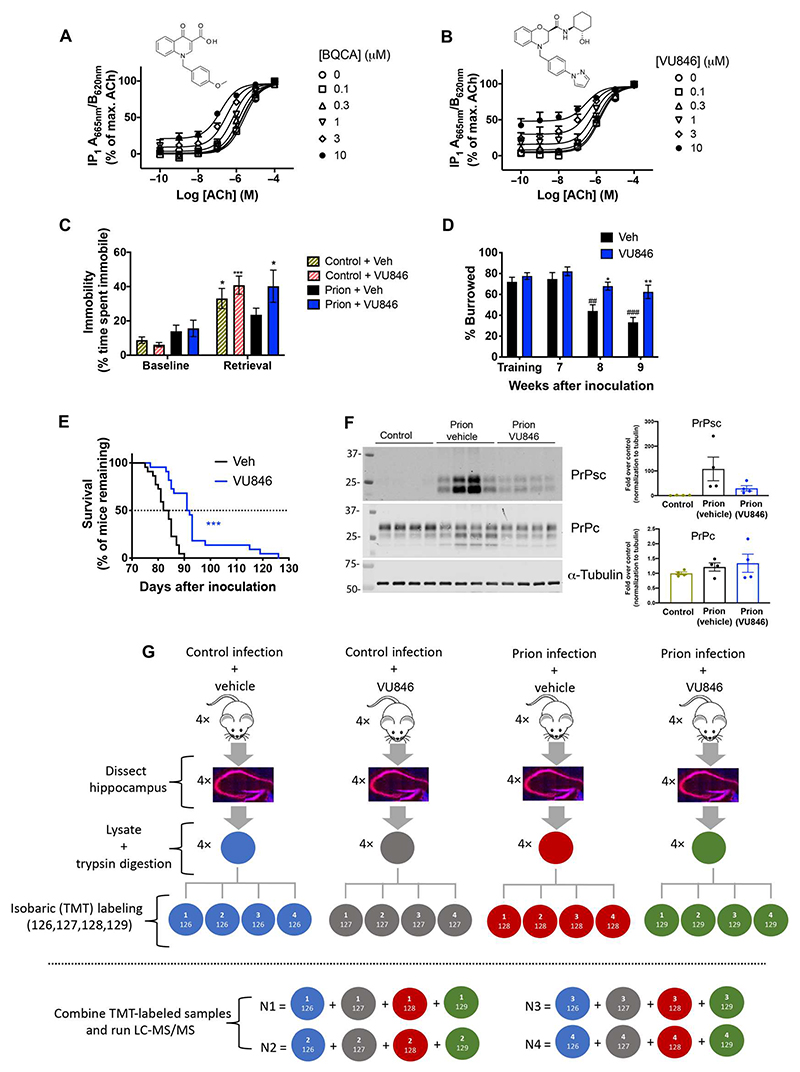
M_1_ receptor PAMs restore learning and memory and prolong survival in murine prion disease. (**A** and **B**) The accumulation of IP1 in mouse cortical primary neuronal cultures treated with acetylcholine (ACh) and the M_1_ receptor PAMs BQCA (A) or VU846 (B). Data are means ± SEM, *n* = 4. VU846 cooperativity with acetylcholine = logαβ = 1.38; *n* = 4. (**C**) Fear conditioning response of control and prion-infected male mice after acute intraperitoneal administration of vehicle (20% Tween 80) or VU846 (10 mg/kg) 30 min before training and retrieval. *n* = 15 to 19 mice per group. Two-way ANOVA with Sidak’s multiple comparisons test, ***P < 0.001 and *P < 0.05 versus control + vehicle. Data shown as means ± SEM. (**D**) Burrowing response of control and prion-infected female mice after administration of vehicle or VU846 as indicated, 30 min before each burrowing session (from 7 w.p.i.). *n* = 12 or 13 mice per group. ##*P* < 0.01 and ###*P* < 0.001 weeks after inoculation versus training; *P < 0.05 and **P < 0.01 prion vehicle versus prion VU846. (**E**) Kaplan-Meier survival plots for prion-infected male and female mice treated with vehicle (*n* = 22; black line) or VU846 (*n* = 22; blue line) as indicated, daily from 7 w.p.i. ***P < 0.001 by Gehan-Breslow-Wilcoxon test. (**F**) Western blot analysis on lysates from control, prion vehicle, and prion VU846 mouse hippocampi. Each lane represents a different mouse (*n* = 4). Bar graphs represent means ± SEM, with individual values that are also displayed. (**G**) Experimental outline and sample preparation for TMT mass spectrometry–based proteomics.

We have previously reported that defective learning and memory associated with a disruption of hippocampal cholinergic innervation in murine prion disease can be restored by orthosteric and allosteric M_1_ receptor ligands (15). This was observed in Tg37 mice, an engineered strain that overexpresses (by three times) murine prion protein, infected with Rocky Mountain Laboratory (RML) scrapie prion-brain homogenate or control normal brain homogenate (NBH) (26). In this system, the M_1_ receptor–selective PAM, BQCA, was shown to be highly tolerated and, after chronic (daily) dosing, prolonged the survival of prion-diseased mice (15). Here, we extended these studies using a next-generation M_1_ PAM, VU846 (28), which, in mouse cortical neuronal cultures, showed high cooperativity with acetylcholine in second-messenger, myoinositol 1 phosphate (IP1) assays (Fig. 1, A and B). In addition, a single administration of VU846 (10 mg/kg) 30 min before fear conditioning training completely restored defective contextual fear conditioning learning and memory in prion-diseased animals (Fig. 1C).

We next tested whether VU846 had the potential to modify the progression of prion disease. In these experiments, animals were dosed daily with VU846 (10 mg/kg) from 7 weeks postinoculation (w.p.i.), a time point at which animals showed the first signs of misfolded PrP^Sc^ (fig. S1, A to C) but no other indicators of disease. Animals treated with vehicle showed reduced performance in burrowing, an innate behavior associated with hippocampal function, whereas burrowing behavior in prion-infected animals treated with VU846 was improved (Fig. 1D). Furthermore, there was a significant delay in the onset of terminal clinical symptoms in animals dosed daily with VU846 (Fig. 1E), with some animals showing markedly extended life spans (Fig. 1E). A slowing in disease progression was further evident in the observed reduction in PrP^Sc^ accumulation in VU846-treated animals (Fig. 1F). In summary, these studies established that the M_1_ PAM, VU846, restored learning and memory in murine prion disease when administered acutely and had disease-modifying properties that corrected for behavioral abnormalities and promoted survival upon chronic administration.

### Molecular markers of neurodegeneration and neuroinflammation were up-regulated in murine prion disease

**Fig. 2:**
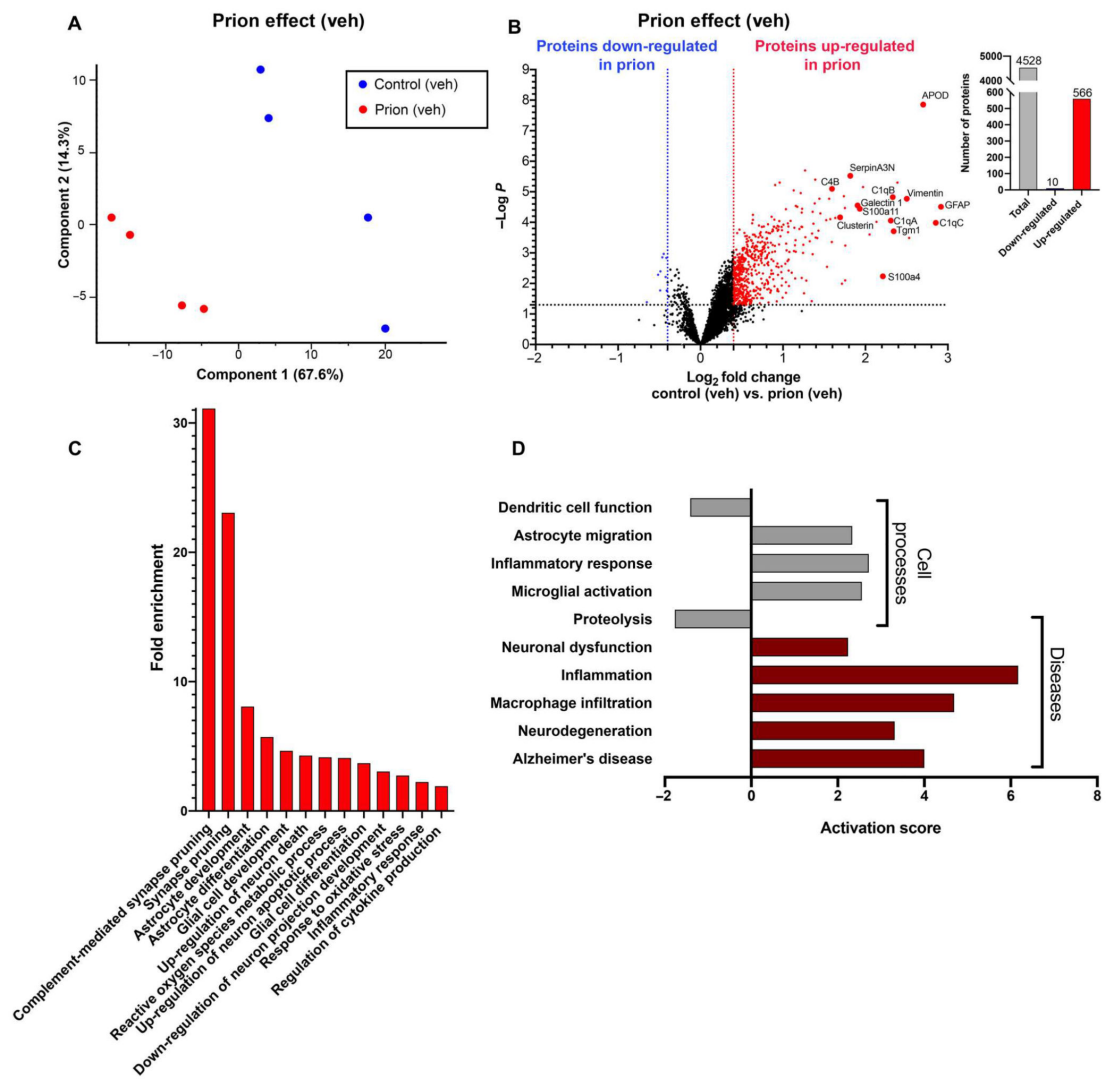
Molecular markers of neurodegeneration and neuroinflammation are up-regulated in a murine prion disease model. (**A**) Principal components analysis (PCA) of the global proteomic study data of four control + vehicle and four prion-infected + vehicle mice. (**B**) Volcano plot showing the differential expression of proteins in the control + vehicle versus prion + vehicle. Red and blue points represent the proteins with significantly increased or decreased expression, respectively [false discovery rate (FDR) < 0.05, ±0.4 log_2_ fold change]. The bar graph shows the total number of proteins analyzed (gray) and the number of proteins that were significantly increased (red) or decreased (blue) (FDR < 0.05, ±0.4 log_2_ fold change). (**C**) Gene ontology (GO) analysis of proteins that were significantly up-regulated in prion + vehicle compared with control + vehicle mice. GO “biological process” terms are plotted against the fold enrichment relative to the expected number of gene lists of these sizes. (**D**) Pathway Studio analysis of “cell processes” (gray) and “diseases” (red) associated with the proteins that are significantly up-regulated in prion + vehicle compared with control + vehicle mice.

We next assessed changes to the proteome caused by prion infection, herein called “prion effect.” This was conducted on hippocampi isolated from Tg37 mice inoculated with RML or, as a control, with NBH at 3 weeks of age and then treated with vehicle intraperitoneally (i.p.) from 7 to 10 w.p.i. (Fig. 1G). Principal components analysis (PCA) showed good separation of proteins of the control animals from the prion-infected animals (Fig. 2A). The total number of proteins identified was 6208, of which 4528 met the robust criteria and were quantified (data file S1, sheet 1). Of these, 566 proteins were significantly up-regulated by more than 0.4 log_2_ fold change in prion-diseased mice, whereas 10 proteins were significantly down-regulated (Fig. 2B and data file S1, sheet 2). Gene ontology (GO) analysis showed that the proteins up-regulated in prion-diseased mice fell into enrichment groups associated with processes known to be up-regulated in human neurodegenerative diseases (data file S1, sheet 3), including neuronal death, synaptic pruning, neurofibrillary tangle assembly, oxidative stress, and protease activity (Fig. 2C). In addition, molecular markers of neuroinflammation and particularly indicators of astrocyte and microglia activation [such as vimentin, galectin-1, and glial fibrillary acidic protein (GFAP)] were up-regulated in murine prion disease (Fig. 2, B and C). The activation of neuroinflammatory pathways was also evident by the up-regulation of components of the neuronal complement system, such as C1qA, C1qB, and C1qC (Fig. 2, B and C). Many of these neuroinflammatory markers are also reportedly up-regulated in human neurodegenerative diseases, including AD (29, 30). This included, for example, the up-regulation of several members of the apolipoprotein family (Fig. 2B and data file S1, sheet 2), including ApoE. This is consistent with reports from human prion disease where ApoE was also reported to be up-regulated (31, 32). Furthermore, key enzymes and transporters, such as transglutaminase 1 (Tgm1) and ATP binding cassette subfamily A member 1 (ABCA1) that have been implicated in the clearance of misfolded protein (33, 34), were up-regulated in murine prion disease (Fig. 2B and data file S1, sheet 2).

Further bioinformatic analysis was conducted using Pathway Studio software that quantitatively assesses proteomic (and transcriptomic) changes with functions reported in the literature to assign an “activation score.” In this analysis, cell processes such as astrocyte migration, microglial activation, and inflammation are seen to have a positive activation score, indicating that these processes are up-regulated in prion disease (Fig. 2D and data file S1, sheet 4). Assessment of a “diseases caused” parameter in this software showed that the proteomic changes associated with murine prion disease are positively correlated with disease indicators for neurodegeneration and neuronal dysfunction (Fig. 2D and data file S1, sheet 5).

### VU846 normalizes brain processes that are dysregulated in prion disease

**Fig. 3:**
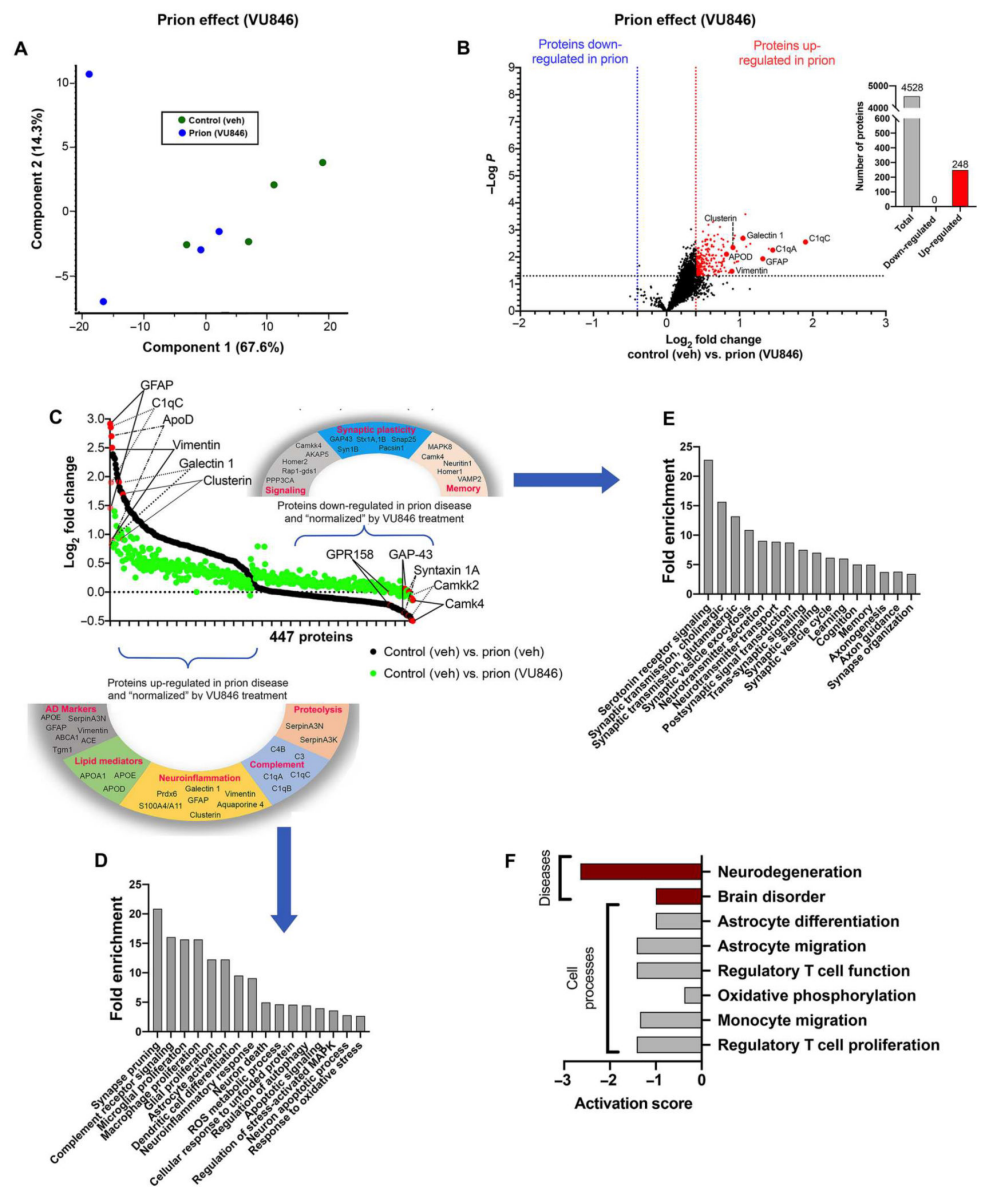
M_1_ receptor PAM VU846 normalizes brain processes that are dysregulated by prion disease. (**A**) Principal components analysis (PCA) of proteomic study data of four control + vehicle and four prion-infected + VU0486 (10 mg/kg)–treated mice. (**B**) Volcano plot representation of differential protein expression in the control + vehicle versus prion + VU846 samples. Red and blue points represent the proteins that were significantly increased or decreased in expression, respectively (FDR < 0.05, ±0.4 log_2_ fold change). Bar graph represents the total number of proteins analyzed (gray) and the number of proteins that were significantly up-regulated (red) or down-regulated (blue) (FDR < 0.05, ±0.4 log_2_ fold change). (**C**) Normalization plot of the 477 proteins that were significantly different (*P* < 0.05) between the prion effect in the context of vehicle and the prion effect in the context of VU846. (**D**) GO analysis of proteins that were significantly up-regulated by a prion effect in the context of vehicle and normalized by a prion effect in the context of VU846. ROS, reactive oxygen species. (**E**) GO analysis of proteins that were significantly down-regulated by a prion effect in the context of vehicle and normalized by a prion effect in the context of VU846. (**F**) Pathway Studio analysis of the overall impact of VU846-mediated normalization of proteins that are either up- or down-regulated in prion disease.

We next wanted to assess the impact of VU846 administration on the proteomic changes seen in prion disease. In these experiments, prion-infected mice were treated daily with VU846 (10 mg/kg, i.p.) from 7 to10 w.p.i., at which time the hippocampi were dissected and analyzed by proteomics. In contrast to hippocampi from prion-diseased animals treated with vehicle, wherein >500 proteins were up-regulated, those from animals treated with VU846 exhibited only 248 proteins that were significantly up-regulated (>0.4 log_2_ fold change; *P* < 0.05) (Fig. 3, A and B, and data file S2, sheet 1). These data suggest an alleviation of prion disease in animals treated with VU846, an observation reflected in PCA, which indicated that there was little variation between the abundance of proteins from noninfected, vehicle-treated animals and that of proteins from prion-infected animals treated with VU846 (Fig. 3A).

Proteins that showed a significantly different abundance in prion-diseased animals (447 proteins; black dots in Fig. 3C) were plotted against the same proteins in prion-diseased animals treated with VU846 (green dots in Fig. 3C and data file S2, sheet 2). This generated what we refer to as a “normalization plot,” wherein the closer the green dots are to zero, the closer VU846 maintained expression of said protein at normal levels (that is, non-disease levels) (Fig. 3C). The results show that proteins that were up- or down-regulated in prion-diseased mice were subsequently normalized—restored to near-normal levels—by VU846 (Fig. 3C).

### VU846 reduced the expression of molecular markers of neurodegeneration

The normalization plot illustrated that markers of microglial and astrocytic activation (such as GFAP, vimentin, clusterin, and galectin-1) as well as components of the complement system (including C1qA, C1qB, and C1qC and the complement receptors C4B and C3) were increased in prion-diseased mice. These same proteins were expressed at lower levels in animals treated with VU846 (Fig. 3C). Furthermore, molecular markers of neurodegeneration, including those shown to be associated with AD such as apolipoproteins ApoE, ApoD, and ApoC (34–36), as well as key enzyme markers such as Tgm1 (37, 38), and regulators of proteolysis such as serpinA3N and serpinA3K (39, 40), were similarly increased in prion disease and reduced by VU846 (Fig. 3C). GO enrichment analysis indicated that the proteins reduced in expression by VU846 fell into groups associated with disease responses such as neuroinflammatory response, apoptotic and neuronal death pathways, reactive oxygen species metabolism, and synapse pruning (Fig. 3D and data file S2, sheet 3). Collectively, these molecular data are consistent with the suggestion that VU846 may reduce the severity of prion disease.

### VU846 increased expression of synaptic proteins in prion disease

Synaptic proteins, including SNAP-25 and syntaxin 1A/1B, as well as signaling proteins, including calcium–calmodulin protein kinase 4 and mitogen-activated protein kinases (such as MAPK8), showed decreased expression in prion disease (Fig. 3C). These proteins are expressed at near-normal levels in prion-diseased animals treated with VU846 (Fig. 3C and data file S2, sheet 4). This suggests that processes identified in the GO analysis to be associated with these proteins—such as synaptic organization, memory, neurotransmitter secretion, and long-term potentiation—are disrupted in prion disease and “normalized” by treatment with VU846 (Fig. 3E and data file S2, sheet 4). The overall impact of VU846-mediated normalization of proteins was assessed using Pathway Studio. This analysis indicated that VU846 decreased (that is, generated a negative activation score) the abundance of markers of inflammation and neurodegeneration (Fig. 3F). Although this remains to be assessed on biological and histopathological levels, these findings further support the conclusion that VU846 may reduce disease severity.

### VU846 modulates the expression of a subset of proteins associated with prion disease

**Fig. 4:**
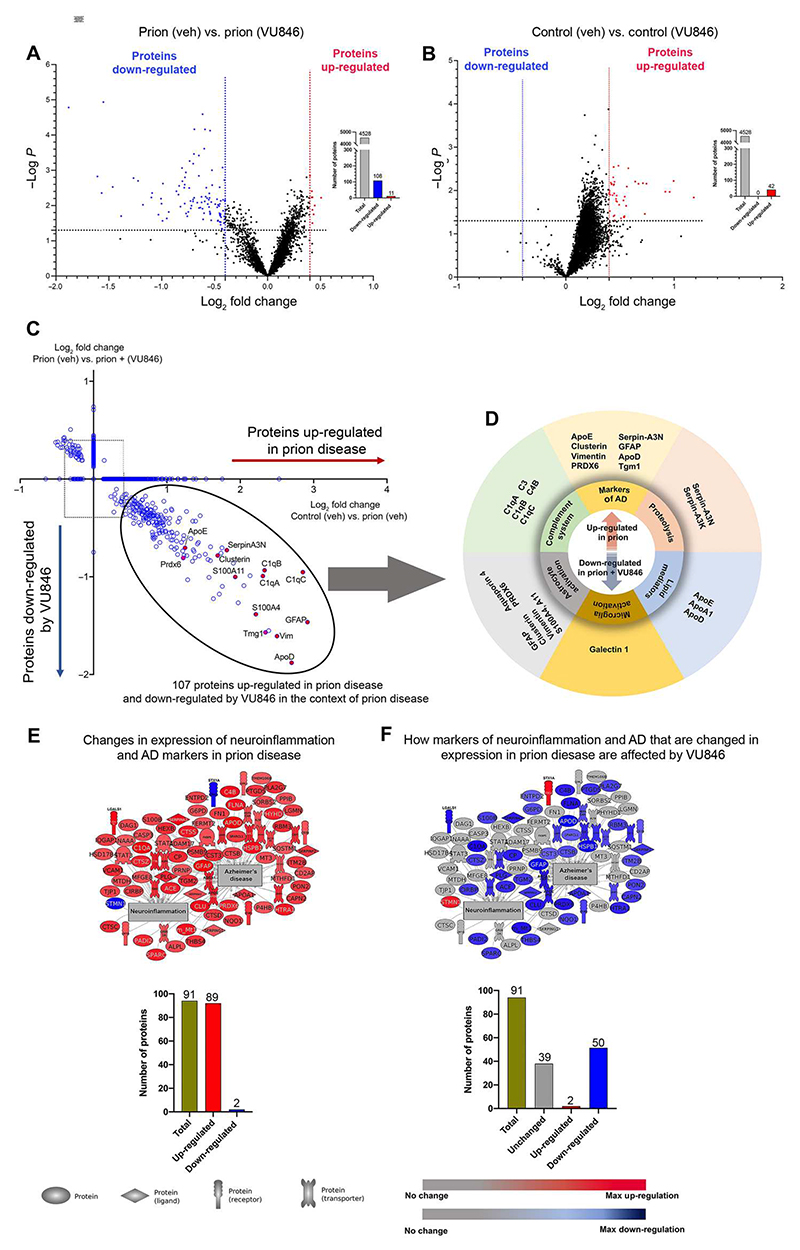
M_1_ receptor PAM VU846 modulates the expression of a subset of hippocampal proteins associated with prion disease. (**A** and **B**) Volcano plots of differential protein expression by the PAM effect in (A) RML prion-diseased mice (prion + VU846 versus prion + vehicle) and (B) control NBH mice (control + VU846 versus control + vehicle). Blue and red points represent the proteins that are significantly decreased or increased in expression, respectively (FDR < 0.05, ±0.4 log_2_ fold change). Bar graph represents the total number of proteins analyzed (gray) and the number of proteins that were significantly down-regulated (blue) or up-regulated (red). (**C**) Quadrant scatterplot showing the effect of VU846 (10 mg/kg) in the context of prion disease. The *x* and *y* axes represent fold changes of proteins that are changed by prion effect and PAM effect, respectively. Proteins outside the square box are significantly changed in expression (FDR < 0.05, ±0.4 log_2_ fold change). (**D**) Grouping of proteins associated with neurodegenerative diseases that are up-regulated in the prion effect and down-regulated in the PAM effect. (**E**) Representative image from Pathway Studio showing 94 proteins from the proteomic dataset whose overall expression levels are associated with AD and neuroinflammation. The bar graph summarizes these changes. (**F**) Effect of VU846 on proteins that are associated with AD.

At this point, we wondered: Of the protein changes seen in prion-diseased mice, how many of these changes are affected by VU846 treatment? To address this question, we first established the impact of VU846 on prion disease by comparing the difference in protein expression between prion-infected mice treated with vehicle and those treated with VU846—we called this comparison the “PAM effect.” In this analysis, 108 proteins were down-regulated by VU846 treatment and 11 were up-regulated (Fig. 4A and data file S2, sheet 5). As a control for these experiments, we assessed changes to the hippocampal proteome in response to VU846 in noninfected animals. Under these conditions, there was very little effect of drug, with only 42 proteins exhibiting statistically significant up-regulation and no proteins showing down-regulation (Fig. 4B and data file S2, sheet 6).

We next established the overlap in the proteins associated with disease (prion effect) with those associated with the action of VU846 in disease (PAM effect) (Fig. 4, C and D, and data file S2, sheet 7). This analysis revealed that of the 94 proteins associated with neuroinflammation/AD in prion disease (prion effect) using Pathway Studio, about half were affected by VU846 (PAM effect) (Fig. 4, E and F). Hence, VU846 appears to mediate a partial correction of the dysregulated protein markers associated with disease (specifically, neuroinflammation and AD), and this likely contributes to the observation that PAM treatment slows but does not completely halt disease progression.

### Validation of the impact of VU846 in prion disease: Biomarkers of disease modification

**Fig. 5:**
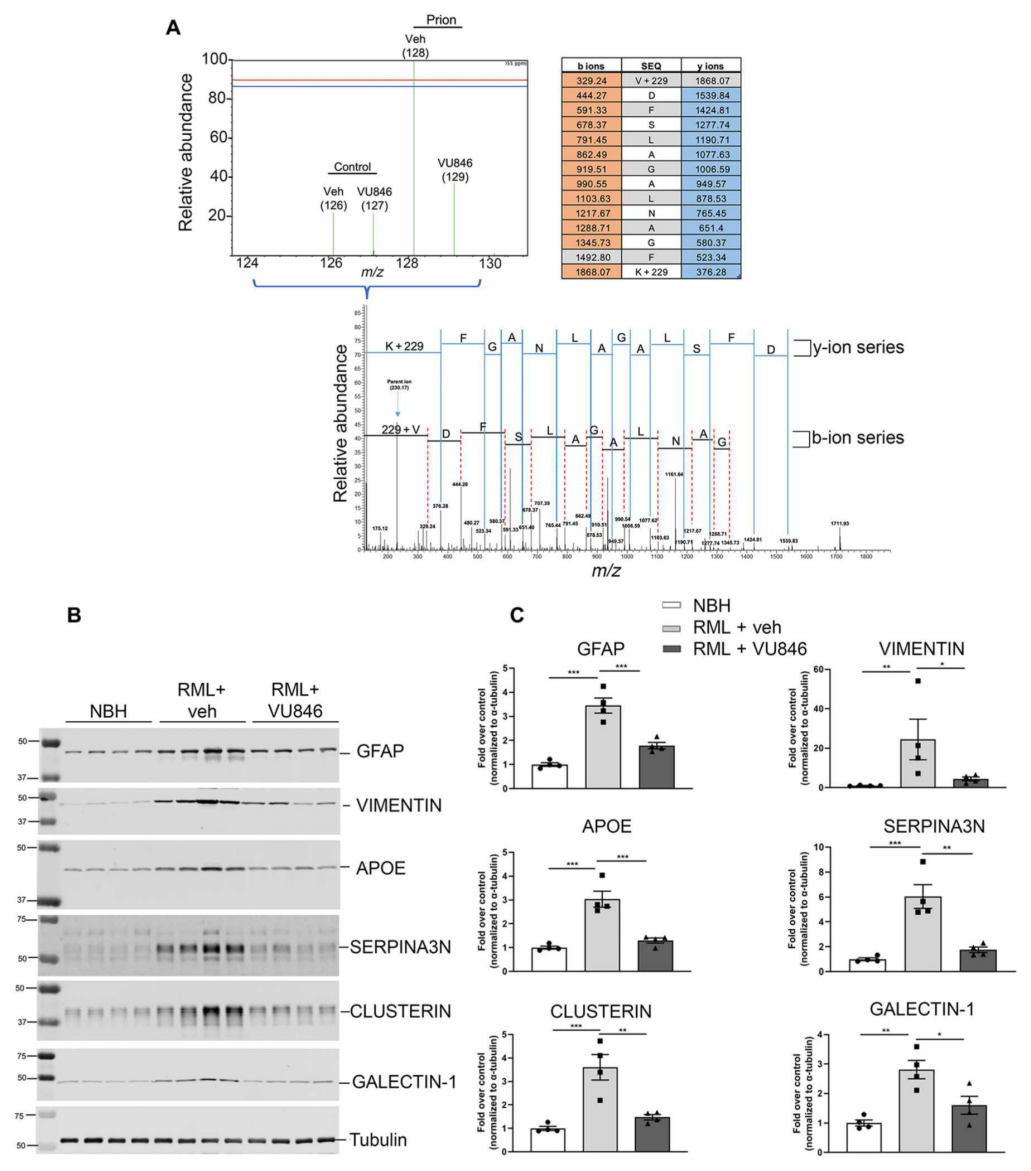
Markers of disease modification modulated by VU846 are validated by Western blotting. (**A**) Representative spectra from the mass spectrometry–proteomic data for the marker of astrocyte and microglial activation, GFAP. *m*/*z*, mass/charge ratio. (**B**) Western blot analysis of hippocampi lysate showing changes in expression of selected markers of astrocyte and microglial activation and of neurodegeneration in the control + vehicle, prion-infected + vehicle, and prion-infected + VU846 (10 mg/kg) mice. Each lane represents a different mouse. (**C**) Quantification of Western blots. Data are means ± SEM; *n* = 4. ***P < 0.001, **P < 0.01, and *P < 0.05 by two-way ANOVA with Sidak’s multiple comparisons test. SEQ, sequence.

We selected key indicators of VU846 activity emerging from the mass spectrometry (MS) analyses to probe further using Western blotting. These consisted of markers of astrocytic and microglial activation (GFAP, vimentin, galectin-1, and clusterin) and indicators of neurodegenerative disease (ApoE and serpinA3N) that each demonstrated significant prion effects and PAM effects from the MS proteomic analysis (Fig. 5A). Consistent with the MS data, all the protein markers tested were up-regulated in expression in prion-diseased animals and were restored to near-normal levels in prion-diseased animals upon treatment with VU846 (Fig. 5, B and C). These experiments not only confirmed the MS data but also established that Western blotting could be used in future studies to probe the disease-modifying properties of M_1_ receptor ligands.

### Different M_1_ PAMs mediate similar effects in prion disease

We previously reported that the prototypical M_1_ PAM, BQCA, restored learning and memory deficits and slowed disease progression in a mouse model of prion disease (15). Therefore, next, we tested whether BQCA had similar effects on the proteome of prion-infected mice as that observed here for VU846. This was a relatively small-scale experiment (further details in Materials and Methods; fig. S2), wherein 2202 proteins qualified for analysis (fig. S3 and data file S2, sheet 8) and of these, 56 were up-regulated by more than 0.4 log_2_ fold and 15 were down-regulated in prion disease (data file S2, sheets 9 and 10). The effect of prion disease was significantly dampened by chronic daily treatment of BQCA from 7 w.p.i. (fig. S3A and data file S2, sheet 11). The proteins regulated by BQCA fell into the same classes of proteins that were regulated by VU846, for example, those involved in neuroinflammation (such as GFAP, vimentin, galectin 1, and clusterin) and markers of neurode-generation, including ApoE, ApoO, S100 proteins, and Prdx6 (fig. S3B and data file S2, sheet 11). Thus, two chemically distinct M_1_ PAMs (BQCA and VU846) similarly affected neuroinflammatory and disease adaptation processes (data file S2, sheet 12).

### Transcriptomic studies support prion disease modifying effects of VU846

**Fig. 6:**
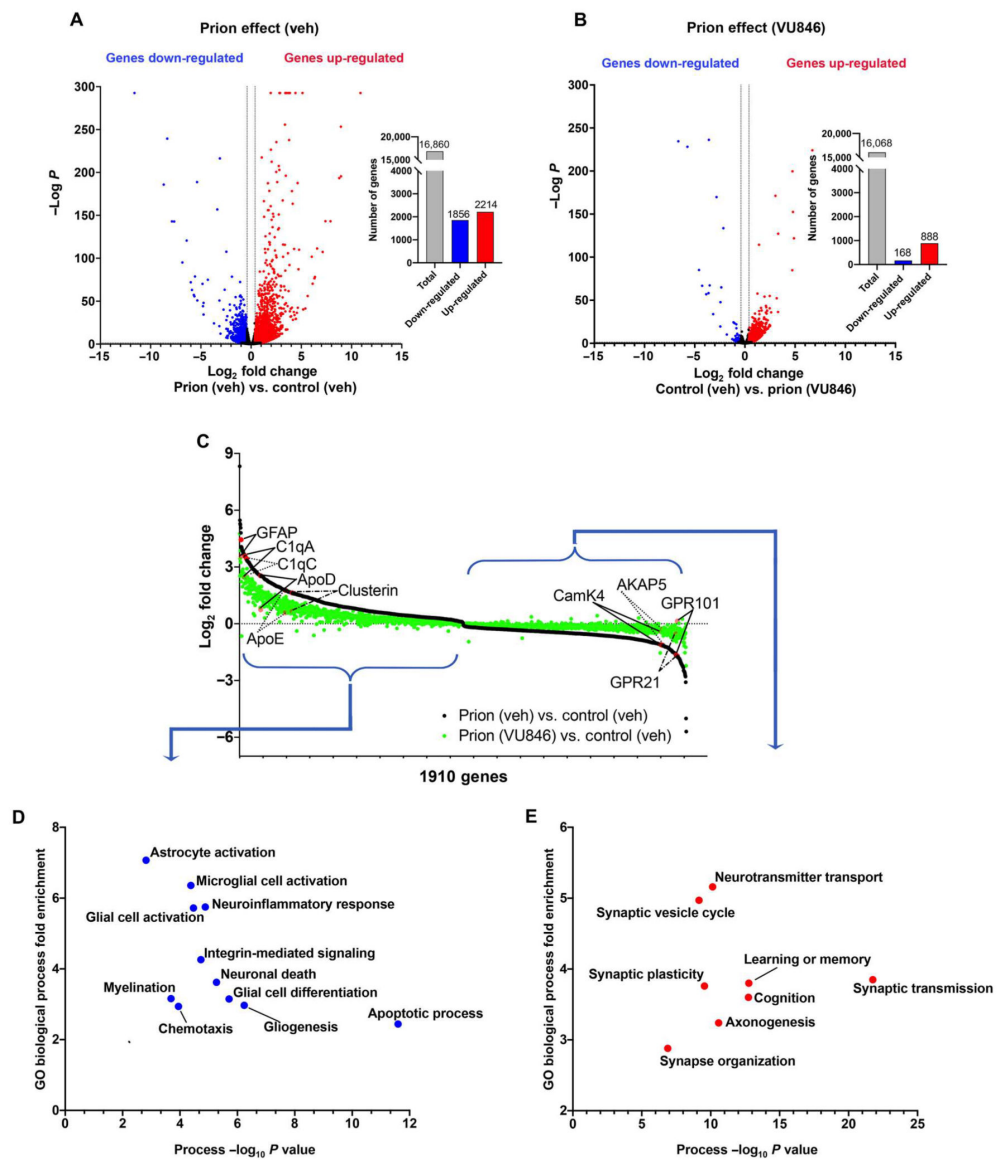
Transcriptomic studies reveal additional changes in transcripts in prion disease that are modified by VU846. (**A** and **B**) Volcano plots of differential gene transcription in the (A) control + vehicle versus prion + vehicle and (B) control + vehicle versus prion + VU846. Red and blue points represent genes with significantly increased or decreased levels of transcript, respectively (FDR < 0.05, ±0.4 log_2_ fold change). (**C**) Normalization plot of the 1910 genes that were significantly different (*P* < 0.05) between prion effect in context of vehicle and prion effect in context of VU846. (**D**) Fisher’s exact test analysis for GO term biological processes of the genes that were significantly up-regulated by a prion effect in the context of vehicle and normalized by a prion effect in the context of VU846. The *x* axis is −log_10_ of the *P* value obtained from the Fisher’s exact test, and the *y* axis is the relative difference between the percentages of significantly differentially transcribed genes that carried the depicted annotations over the percentage of all sites that carried the same annotation. (**E**) The Fisher’s exact test for GO term biological processes the genes that were significantly down-regulated by the prion effect in the context of vehicle and normalized by the prion effect in the context of VU846.

To complement the proteomic studies, we conducted a global transcriptomic analysis of hippocampi derived from animals treated daily with vehicle or VU846 from 7 to 11 w.p.i. (fig. S4A). In these experiments, prion disease resulted in >1800 gene transcripts to be down-regulated and >2200 genes to be up-regulated by more than 0.4 log_2_ fold (Fig. 6A and data file S3, sheets 1 to 3). Treatment with VU846 substantially dampened the transcriptional changes associated with disease (Fig. 6B and data file S3, sheet 4). Thus, in animals treated with VU846, only 168 genes were down-regulated and 888 were up-regulated (Fig. 6B). VU846 had little impact on the transcriptome in nondiseased controls (fig. S5 and data file S3, sheet 5).

In analysis similar to that conducted in the proteomic study, we constructed a normalization plot to assess the impact of VU846 on transcription in the context of prion disease (Fig. 6C and data file S3, sheet 6). GO analyses of these data established that markers of neuroinflammation and genes associated with neuronal death and apoptosis that were up-regulated in prion disease were significantly reduced by VU846 (Fig. 6D and data file S3, sheet 7). Conversely, the expression of genes that were down-regulated in prion disease was significantly increased by VU846 (Fig. 6C, right). These genes were enriched in brain processes that are known to be disrupted in neurodegenerative disease, including synaptic plasticity, learning and memory, cognition, and synaptic transmission (Fig. 6E and data file S3, sheet 8). Overall, these transcriptional data are consistent with the proteomic studies in establishing that treatment with VU846 corrects, or normalizes, dysregulated brain processes associated with prion disease.

### VU846 modifies the expression of a subset of genes associated with prion disease

**Fig. 7:**
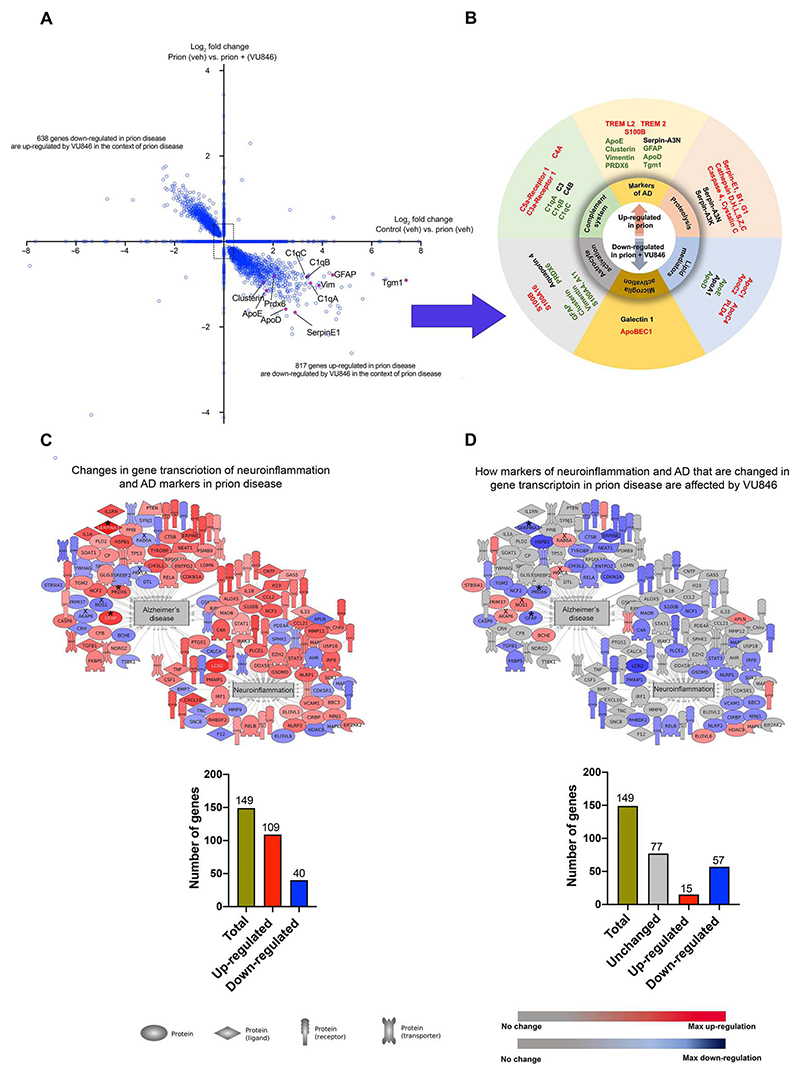
M_1_ receptor PAM, VU846, has a PAM effect on the prion mouse hippocampal transcriptome. (**A**) Quadrant scatterplot showing the effect of VU846 (10 mg/kg) on gene transcription in the context of prion disease. The *x* and *y* axes represent fold changes of genes that are changed by prion and PAM effects, respectively. Genes lying outside of the square box are significantly changed (FDR < 0.05, ±0.4 log_2_ fold change). (**B**) Grouping of genes associated with neurodegenerative disease that are up-regulated in the prion effect and down-regulated in the PAM effect. (**C** and **D**) Representative images generated with Pathway Studio showing the link between VU846 treatment and regulation of neuroinflammation and markers of AD. A proportion of the genes that are up- or down-regulated in the prion effect are subsequently affected by VU846 in the PAM effect.

As described above for the proteomic analysis, we next assessed whether VU846 affected a specific set of disease-associated genes. We did this by comparing all the changes associated with disease (that is, the prion effect) with the changes mediated by VU846 in the context of disease (that is, the PAM effect). In this analysis, 817 genes were transcriptionally up-regulated in the prion effect and subsequently down-regulated in the PAM effect (Fig. 7A, bottom right, and data file S3, sheet 9). Among these were genes associated with the complement system and microglia and astrocyte activation and included genes that were similarly regulated in the proteomic analysis described above (green text in Fig. 7B). There was a similar overlap between the proteomic and transcriptomic datasets in molecular markers of neurodegeneration, proteolysis, and lipid mediators (Fig. 7B). Not all transcriptional changes were linked with corresponding proteomic changes (data file S4, sheets 1 to 4), suggesting that the impact of VU846 on protein levels may, in some instances, lie beyond transcription and might be due to regulation of translation or protein degradation.

The overall outcome of these analyses was that only a proportion of the genes that were changed in prion-diseased mice were subsequently affected by VU846 (Fig. 7, C and D). This is illustrated by Pathway Studio analysis of the genes classified as associated with neuroinflammation and/or AD, where only 48% of the genes that showed changes with prion disease were affected by VU846 treatment (Fig. 7, C and D).

## Discussion

All attempts to identify a therapy that can substantially delay the progression of neurodegenerative disease, including AD, have thus far failed in preclinical development or clinical trials (6–8). The emergence of an array of AD risk factors from genome-wide association studies (11, 41, 42) has provided a number of potential targets that are distinct from the extensively tested β- and γ-secretase inhibitors and antibodies that target Aβ (6–8). However, the paucity of knowledge in terms of how these proteins operate in the context of neurodegenerative disease, as well as the intractability of many of these as pharmacological targets, has limited preclinical validation and subsequent drug discovery efforts (43, 44). Here, we describe how targeting the M_1_ receptor with PAMs that amplify the spatial and temporal patterns of physiological stimulation of M_1_ receptors by acetylcholine can reduce the molecular markers of neuroinflammation and adaptive processes associated with prion-mediated neurodegeneration. Our proteomic and transcriptomic data further indicate that such M_1_ receptor activation may be critical for maintaining synaptic function and mitochondrial/redox homeostasis. Thus, our study provides support for the M_1_ receptor as an attractive therapeutic target to potentially reduce neurodegenerative disease severity and maintain synaptic function, thereby increasing lifespan and maintaining neurological function.

The muscarinic receptor family members were among the first G protein–coupled receptors to be cloned and characterized and, hence, are considered by many as prototypical (45). The ensuing decades of research have resulted in a rich understanding of the signaling, pharmacology, and physiological roles of this receptor family (46, 47). The M_1_ receptor in particular, with its high expression in memory centers and procognitive properties, has been proposed as a target for treating memory loss in AD that would avoid the dose-limiting, adverse responses associated with current clinically approved cholinesterase inhibitors (24, 25). The challenge has been to develop M_1_ receptor–selective drugs, because the orthosteric acetylcholine binding site is highly conserved across the five muscarinic receptor subtypes (21). Hence, orthosteric ligands (such as xanomeline) have failed clinically as AD therapeutics primarily due to cholinergic adverse responses mediated by peripheral M_2_ and M_3_ receptors (22). An alternative strategy has been to target the allosteric pocket through ligands that act in a receptor subtype-selective manner to enhance receptor activity cooperatively with the natural ligand acetylcholine (25). The prospect that these PAMs might offer an approach to treating memory loss in AD through the restoration of cholinergic transmission has led to an expansion of PAM chemotypes displaying various pharmacological profiles (19, 48, 49). This has allowed interrogation of the preclinical pharmacology of PAMs with distinct levels of intrinsic efficacy, biased agonism, and levels of cooperativity with the physiological agonist acetylcholine. These studies led to the conclusion that M_1_ receptor PAMs that display moderate to high levels of cooperativity with acetylcholine while having low intrinsic efficacy and no ligand bias would provide procognitive effects with few associated cholinergic adverse responses (19, 50). VU846 shows many of these favored characteristics and, together with good brain penetration and favorable drug metabolism and pharmacokinetics properties (28), makes for an excellent proof-of-concept M_1_ receptor–selective PAM to broadly assess the preclinical benefits of this class of pharmacological agent.

Murine prion disease is associated with a disruption in hippocampal cholinergic innervation that results in defective learning and memory that can be restored with clinically approved cholinesterase inhibitors and the orthosteric muscarinic agonist xanomeline (15). We reasoned, therefore, that murine prion disease was a good model to investigate therapeutic approaches to restoring defective cholinergic transmission and subsequently demonstrated that the M_1_ PAM, BQCA, could similarly restore defective learning and memory in this mouse model (15). What was not clear from these previous studies was whether prion disease exhibited further molecular profiles that overlapped with human neurodegenerative disease, including AD, that would allow for a more extended application of this model in preclinical assessment. Using global proteomic and transcriptomic analyses, we established here that the molecular profile of murine prion disease that includes neuroinflammation, markers of mitochondrial dysfunction, and increased oxidative stress does show substantial overlap with AD and other forms of human neurodegenerative disease (51–55). In this way, our study indicates that the disease-adaptive changes potentially associated with compensatory mechanisms in human neurodegenerative disease are also operating in murine prion disease. Our study therefore supports the notion that neurodegenerative diseases that are the result of prion-like spreading and propagation of misfolded protein share many common adaptive and molecular features (2, 56).

Given this commonality between murine prion disease and human neurodegenerative disease, it is particularly noteworthy that chronic treatment with the M_1_ PAM VU846 resulted in a significant reduction in the molecular markers of neuroinflammation and neurodegeneration in a manner that correlated with a prolonged survival and maintenance of normal mouse behavior. Dosing of VU846 commenced at a disease stage where molecular markers of disease (such as accumulation of misfolded PrP^Sc^) were already evident, indicating that VU846 was effective after disease had been established. Nonetheless, not all markers were restored to near-normal levels by VU846 treatment, raising the possibility that selective pathways of neuroinflammation and neuronal adaptation and survival may be directly regulated by M_1_ receptor activity.

Last, a notable feature of VU846 activity was that there was little effect of the compound in normal, nondiseased animals. Hence, it is in a disease context where VU846 has the most profound impact on the proteome and transcriptome. This correlates well with previous reports where M_1_ PAMs reportedly had little behavioral effect in control animals, and it was only when there was a disruption in cholinergic transmission mediated by pharmacological intervention or by neurological disease that M_1_ PAMs were seen to have an impact (15, 28, 57).

In conclusion, our study provides mechanistic insight into the observed slowing of disease progression and maintenance of normal behavior mediated by the administration of M_1_ PAMs, specifically those (such as VU846) that display low intrinsic activity but high cooperativity with the natural ligand. The findings support the proposal that M_1_ PAMs might not only be an effective therapeutic strategy to treat memory loss in neurodegenerative diseases, such as AD, but might also be neuroprotective.

## Materials and Methods

### Animal maintenance

The mice were fed ad libitum with a standard mouse chow. The MloxP Tg37 transgenic mice that overexpress mouse PrP^C^ as described in previous studies (15, 58) were provided by G. Mallucci (University of Cambridge Dementia Research Institute). All animal work and care were carried out under a project license according to U.K. Home Office Regulations.

### Inoculation of Tg37 mice with prion

Tg37 hemizygous mice at 3 to 4 weeks were inoculated by intracerebral injection into the right parietal lobe with about 20 μl of 1% (w/v) brain homogenate of RML scrapie prion as described previously (15, 26). Control Tg37 mice were inoculated in a similar manner with about 20 μl of 1% NBH.

### Survival studies

Male and female Tg37 mice were inoculated with RML or NBH as described above. The mice were given intraperitoneal injection of vehicle (20% Tween 80), BQCA (15 mg/kg), or VU846 (10 mg/kg) daily from 7 w.p.i.. Video recordings of the mice were taken every 3 days from 7 w.p.i. The mice were examined daily for early indicators and confirmatory signs of scrapie prion disease, and animals were culled when they developed clinical signs of scrapie. Early indicators include clasping of hind legs when mice are lifted by the tail, unsustained hunched posture, rigid tail, mild loss of co-ordination, piloerection, and being subdued. Confirmatory signs include sustained hunched posture, ataxia, dragging of limbs, significantly abnormal breathing, and impaired writhing reflex. The presence of two early indicator signs plus one confirmatory sign or two confirmatory signs alone was indication of clinical disease.

### Fear conditioning learning and memory test

The fear conditioning experiments were conducted on male mice at 9 w.p.i. with RML or NBH, before the appearance of clinical symptoms. Mice were acclimatized to the behavioral room overnight before the day of the test. M_1_ PAM VU846 (10 mg/kg) or vehicle was administered via intraperitoneal injection on the day of the behavioral test, 30 min before the training. For fear conditioning, mice were placed in the conditioning chamber (Stoelting ANY-maze Fear Conditioning System, Dublin) and allowed to adapt to the chamber for 2 min. The mice received three tone/foot shock pairings, where the foot shock [unconditioned stimulus (US); 2 s, 0.4 mA] always coterminated with a tone [conditioned stimulus (CS); 2.8 kH, 85 dB, 30 s]. The CS-US pairings were separated by 1-min intervals. After completion of the training, the mice remained in the conditioning chamber for 1 min and were then returned to their home cages. The next day, the mice were placed back in the conditioning chamber, and time spent immobile was recorded for 3 min to assess context-dependent learning. The data were analyzed using ANY-maze software (Stoelting, Dublin).

### Burrowing

Assessment of burrowing activity was conducted on female mice from 7 to 9 w.p.i. A day before the burrowing test, mice were placed into individual burrowing cages containing an empty burrowing tube for a 2-hour period to acclimatize. The burrowing tube is a clear, acrylic tubing with one end sealed with transparent plastic. On the test day, mice received vehicle (20% Tween 80) or VU846 (10 mg/kg) via intraperitoneal injection 30 min before the burrowing test. The mice were placed into individual burrowing cages containing a burrowing tube filled with 140 g of food pellets for 2 hours. The amount of food pellets remaining after the 2 hours was weighed, and the burrowing activity was calculated by subtracting the weight of food pellets remaining from the starting weight and expressing the proportion of food pellets that had been displaced as a percentage. The mice were returned to their home cages, and the experiment was repeated on a weekly basis.

### Cortical neuronal primary cultures

Cortical neurons were isolated from 16-day-old embryos of C57BL/6 mice. Dissected brains were immediately placed in ice-cold dissection buffer (Dulbecco’s modified Eagle’s medium), and the cerebral cortices were isolated under a dissecting microscope. Cortex tissues were then mechanically triturated, and cells were resuspended in Hanks’ balanced salt solution, followed by centrifugation at 500*g* for 5 min. The pellets were resuspended in warm Neurobasal medium supplemented with B-27, l-glutamine, and 1% penicillin/streptomycin. The primary cells were plated at a density of 60,000 cells per well in a 96-well microplate that had been precoated with poly-_D_-lysine (50 μg/ml) and maintained at 37°C in a 5% CO_2_ humidified atmosphere. In vitro assays were performed 1 week later.

### IP1 accumulation assay

Cultured mouse embryonic cortical neuronal cells were washed and incubated in 80 μl of 1× stimulation buffer [10 mM Hepes, 1 mM CaCl_2_, 0.5 mM MgCl_2_, 4.2 mM KCl, 146 mM NaCl, 5.5 mM glucose, and 50 mM LiCl (pH 7.4)] for 1 hour at 37°C before drug treatments. Ten microliters of 10× concentrated M_1_ PAM (BQCA or VU846) was added to respective wells in the microplate, followed by 10 μl of 10× concentrated acetylcholine, and incubated at 37°C for 1 hour. The stimulation buffer was removed, and cell lysis buffer (IP-One assay kit, CisBio) was added (40 μl per well) and incubated for 10 min with shaking at 600 rpm. The cell suspensions (7 μl per well) were added to 384-well white ProxiPlates and centrifuged briefly. IP1-d2 conjugate and the anti-IP1 cryptate Tb conjugate (IP-One Tb assay kit, CisBio) were diluted 1:40 in lysis buffer, and 3 μl of each was added to each well. The plate was incubated at 37°C for 1 hour, and fluorescence resonance energy transfer between d2-conjugated IP1 (emission at 665 nm) and Lumi4-Tb cryptate conjugated anti-IP1 antibody (emission at 620 nm) was detected using an Envision plate reader (PerkinElmer). Results were calculated from the 665/620-nm ratio and normalized to the maximum response stimulated by acetylcholine.

### Hippocampal lysate preparation and Western blot analysis

Fresh-frozen hippocampi from RML- and NBH-inoculated mice were transferred into microcentrifuge tubes containing 300 μl of radioimmunoprecipitation assay buffer [50 mM tris-HCl, 1 mM EDTA, 1 mM EGTA, 1% (v/v) Triton X-100, and 0.1% (v/v) 2-mercaptoethanol (pH 7.5)] and sonicated three times for 15 s each at 3- to 5-μm amplitude. The lysate was incubated at 4°C for 2 hours with end-to-end rotation and then centrifuged at 15,000*g* for 10 min at 4°C. The supernatant was transferred into new tubes, and protein concentration was determined using the bicinchoninic acid assay (BCA) protein assay kit according to the manufacturer’s instructions (Thermo Fisher Scientific). Ten micrograms of protein was added to equal volume of 2× Laemmli sample buffer and heated at 95°C for on a 12% SDS–tris-glycine polyacrylamide gel. The proteins were transferred onto a nitrocellulose membrane, blocked in 5% (w/v) fat-free milk, and then immunoblotted with respective primary antibodies (table S1) overnight at 4°C. After washes and incubation with LI-COR IRDye secondary antibody (LI-COR, Cambridge, UK), the proteins were visualized and quantified using the Empiria Studio software (LI-COR). The intensity of the proteins was normalized to the intensity of α-tubulin.

### Proteinase K digestion

For proteinase K (PK) digestion analysis, equal volumes of PK (20 μg/ml) and 40 μg of protein lysate were mixed and incubated at 37°C for 10 min. The digestion reaction was stopped by adding Laemmli sample buffer and heating at 95°C for 5 min. The proteins were separated by electrophoresis on a 12% SDS–tris-glycine polyacrylamide gel, subsequently transferred onto a nitrocellulose membrane, and then blocked in 5% (w/v) fat-free milk. The membrane was immunoblotted with primary antibody to prion protein (Abcam, ab61409) overnight at 4°C, followed by washes and incubation with LI-COR IRDye secondary antibody (LI-COR, Cambridge, UK), and the proteins were visualized and quantified using the Empiria Studio software (LI-COR). The intensity of the proteins was normalized to that of α-tubulin.

### Prion cohorts for proteomic and transcriptomic analysis

For the BQCA proteomic cohort, male and female mice (NBH and RML) were treated (intraperitoneally) with vehicle (5% glucose) or BQCA (15 mg/kg) daily from 7 w.p.i. for 2 weeks. Animals were culled and hippocampus was dissected.

For the VU846 proteomic and transcriptomic cohort, the NBH and RML mice (male and female) were treated intraperitoneally with vehicle (20% Tween 80) or VU846 (10 mg/kg) daily from 7 to 11 w.p.i. Animals were culled, and the hippocampus was dissected from each. The hippocampus from one brain hemisphere was processed for MS-based proteomics, and the other half was processed for transcriptomic analysis.

### Hippocampal preparation for tandem mass tag (TMT) liquid chromatography–tandem MS

The mice were euthanized by cervical displacement, and the brain was removed from the skull and dissected immediately. The hippocampi and cortices were flash-frozen on dry ice. The frozen hippocampi (from one hemisphere of the brain) were transferred into microcentrifuge tubes containing SDS lysis buffer [50 mM triethylammonium bicarbonate (TEAB) and 10% SDS (pH 7.55)] supplemented with protease and phosphatase inhibitors and homogenized using a motorized pellet pestle for 30 s. A total of 20% (w/v) CHAPS and 10% (v/v) NP-40 were added to final concentrations of 2 and 1%, respectively, and the lysate was sonicated three times for 15 s each at 3- to 5-μm amplitude and then centrifuged at 15,000*g* for 10 min at 4°C. The supernatant was transferred into new microcentrifuge tubes, and the protein concentration was determined using the BCA protein assay kit according to the manufacturer’s instructions (Thermo Fisher Scientific). The lysates were normalized to the same protein concentrations (0.8 mg) with SDS lysis buffer to a final volume of 500 μl. The proteins were reduced using 20 mM dithiothreitol at 37°C for 1 hour, followed by alkylation in the dark for 30 min with 100 mM iodoacetamide. The samples were acidified with 12% phosphoric acid to a final concentration of 1.2% (v/v) and then digested overnight at 37°C with sequence grade trypsin at a trypsin-to-protein ratio of 1:20 (w/w) using the ProtiFi S-Trap midi digestion columns (ProtiFi, Huntington). Eluted peptides were dried in a vacuum concentrator, resuspended in 0.1% trifluoroacetic acid (TFA), and desalted using Pierce peptide desalting columns (Thermo Fisher Scientific). The eluted peptides were dried, resuspended in 50 mM Hepes buffer (pH 8.5), and labeled with TMTsixplex (Thermo Fisher Scientific) at 25°C for 2 hours with orbital shaking at 500 rpm. The TMT-to-peptide ratio was 2.5:1. The labeling reaction was quenched with 5% (v/v) hydroxylamine at a final concentration of 0.4% at 25°C for 30 min with orbital shaking at 500 rpm. The peptides were dried, resuspended in 0.1% TFA, and separated into 10 fractions using the Pierce high-pH reversed-phase fractionation columns (Thermo Fisher Scientific). The eluted fractions were dried and resuspended in 0.1% formic acid for liquid chromatography–tandem MS (LC-MS/MS) analysis.

For the VU846 cohort, hippocampi from mice in each of the four experimental groups (control + vehicle, control + VU846, prion + vehicle, and prion + VU846) were processed, labeled with respective TMT, combined, and analyzed by LC-MS/MS to give one experimental run. This process was repeated with the other three mice in each experimental group to give four independent runs and datasets. For the BQCA cohort, hippocampi from three mice in each of the experimental groups were pooled together, processed, and labeled with respective TMT, combined, and analyzed by LC-MS/MS.

### TMT LC-MS/MS and data processing

Samples were analyzed by using an LTQ Orbitrap Velos mass spectrometer (Thermo Fisher Scientific) equipped with an ultrahigh-pressure LC system (RSLCnano). The samples were loaded at high flow rate onto a reversed-phase trap column (0.3 mm internal diameter × 1 mm) containing 5-mm C18 300-Å Acclaim PepMap medium (Dionex) maintained at 37°C. The loading buffer was 0.1% formic acid/0.05% TFA/2% acetonitrile (ACN). The peptides were eluted from the reversed-phase trap column at a flow rate of 0.3 μl/min and passed through a reversed-phase PicoFrit capillary column (75 μm i.d. × 400 mm) containing Symmetry C18 100-Å medium (Waters) that was packed in-house using a high-pressure device (Proxeon Biosystems). Peptides were eluted over a period of 4 hours, with the output of the column sprayed directly into the nano-spray ion source of the LTQ Orbitrap Velos mass spectrometer. The LTQ Orbitrap Velos mass spectrometer was set to acquire a one-microscan Fourier transform mass spectrometer (FTMS) scan event at 60,000 resolutions over the mass/charge ratio range of 300 to 2000 Da in positive ion mode. The maximum injection time for MS was 500 ms, and the automatic gain control (AGC) target setting was 1 × 10^6^. Accurate calibration of the FTMS scan was achieved using a background ion lock mass for C_6_H_10_O_14_S_3_ (401.922718 Da). Subsequently, up to 10 data-dependent higher-energy collision dissociation MS/MS were triggered from the FTMS scan. The isolation width was 2.0 Da, with a normalized collision energy of 42.5. Dynamic exclusion was enabled. The maximum injection time for MS/MS was 250 ms, and the AGC target setting was 5 × 10^4^.

The raw data file obtained from each LC-MS/MS acquisition was processed using Proteome Discoverer (version 2.5.0.400, Thermo Fisher Scientific), searching each file in turn using Mascot (version 2.7.07, Matrix Science Ltd.) against the UniProtKB-SwissProt database. The peptide tolerance was set to 10 parts per million, and the MS/MS tolerance was set to 0.02 Da. A decoy database search was performed. The output from Proteome Discoverer was further processed using Scaffold Q + S (version 4.11.0, Proteome Software). Upon import, the data were searched using X!Tandem (Global Proteome Machine Organization). PeptideProphet and ProteinProphet (Institute for Systems Biology) probability thresholds of 95% were calculated from the decoy searches, and Scaffold was used to calculate an improved 95% peptide and protein probability threshold based on the data from the two different search algorithms.

### Analysis of proteomic data

The data were uploaded in Microsoft Excel (version 2016), Perseus (version 1.6.12.0), and Scaffold (version 4.11.0) analytical suites for downstream analysis. For ease of data handling, all data entries were transformed into log_2_ scale and normalized. For a protein to be included in the analysis, the peptides corresponding to the protein must be present in at least three of the four independent datasets. Contaminants, reverse hits, and proteins “only identified by site” were excluded from the analysis.

Statistical analyses were performed using two-tailed Student’s *t* test, one-way analysis of variance (ANOVA), or two-way ANOVA. Significance was defined as *P* < 0.05. All statistical tests were performed using GraphPad Prism software. Graphs were plotted using Perseus, Microsoft Excel, and GraphPad Prism software.

### Hippocampal preparation for transcriptomics

Three mice from each of the four experimental groups (NBH vehicle, NBH VU846, RML vehicle, and RML VU846) were processed for transcriptomic analysis. RNA from each group was extracted using the RNeasy Plus Mini Kit (QIAGEN, Manchester) following the manufacturer’s instructions. The tissue was homogenized in the RNA kit buffer by sonicating three times for 15 s each at 3- to 5-μm amplitude and then centrifuged at 10,000*g* for 3 min at 4°C. The homogenized sample was transferred into the purification columns for RNA purification. RNA was eluted with ultrapure water and concentration, and purity was measured with the Nano-Drop 1000 Spectrophotometer (Thermo Fisher Scientific).

### mRNA library construction and data analysis

RNA samples were processed at the Glasgow Polyomics Research Facility. Each sample was subjected to mRNA polyadenylate enrichment before libraries were generated with a TruSeq Stranded mRNA sample preparation kit (Illumina). The libraries were sequenced paired-end (2 × 75 base pairs) on the NextSeq500 instrument (Illumina) to an average of at least 33 million reads. Raw counts were then converted into FastQC format.

The Galaxy bioinformatics data analysis platform (version 0.72) was used to process raw data FastQC files. The data were analyzed to remove both the TruSeq3 adaptors used for sequencing and the bad-quality RNA sequences using the Trimmomatic flexible read trimming tool for Illumina next-generation sequencing data (Galaxy version 0.36.5). Transcripts showing eight hit-reads matching any adaptor were trimmed off and discarded, and the sliding window trimming function was applied to eliminate bad-quality RNA sequences with a cutoff of 25 Phred. The remaining sequences were mapped to the “mouse-mm10” genome using HISAT2, a fast and sensitive alignment program (Galaxy version 2.1.0), and processed with the StringTie function to assemble and quantify the sequences associated for each gene (BAM files).

Differential gene expression comparisons were performed with “BAM” files using the DESeq2 statistical tool (parametric fit type) on the Galaxy bioinformatic platform. Gene differential expression data were analyzed with two online software suites, GO Panther (www.pantherdb.org) and Pathway Studio (www.pathwaystudio.com), to identify diseases, cell processes, and pathways associated with genes affected by prion and drug.

### Raw data accession codes

All the TMT MS data, RAW files together with the MaxQuant outputs, have been uploaded to PRIDE (project accession: PXD025561). The raw transcriptomic data have been deposited in the Gene Expression Omnibus repository (GEO accession number: GSE202275).

## Supplementary Material

Supplementary Materials

## Data Availability

All the TMT MS data, RAW files together with the MaxQuant outputs, have been uploaded to PRIDE, project accession number PXD025561. The raw transcriptomic data have been deposited in the Gene Expression Omnibus repository, GEO accession number GSE202275. All other data needed to evaluate the conclusions in the paper are present in the paper or the Supplementary Materials. All data are further available from the corresponding authors or through the University of Glasgow’s online data repository.
