# Supplementary material for: M1 muscarinic receptor activation reduces the molecular pathology and slows the progression of prion-mediated neurodegenerative disease
Source: Sci Signal. Author manuscript; Available in PMC 2024 Jul 11. (PMC7616172; doi:10.1126/scisignal.abm3720)
Supplement: Supplementary Materials [file EMS197015-supplement-Supplementary_Materials.zip › scisignal.abm3720_sm.pdf]

Science Signaling
AAAS

Supplementary Materials for

M_1 muscarinic receptor activation reduces the molecular pathology and slows the progression of prion-mediated neurodegenerative disease

Louis Dwomoh *et al.*

Corresponding author: Andrew B. Tobin, andrew.tobin@glasgow.ac.uk;
Sophie J. Bradley, sophie.bradley@soseiheptares.com

Sci. Signal. **15**, eabm3720 (2022)
DOI: 10.1126/scisignal.abm3720

The PDF file includes:

Figs. S1 to S5
Table S1
Legends for data files S1 to S4

Other Supplementary Material for this manuscript includes the following:

Data files S1 to S4
MDAR Reproducibility Checklist

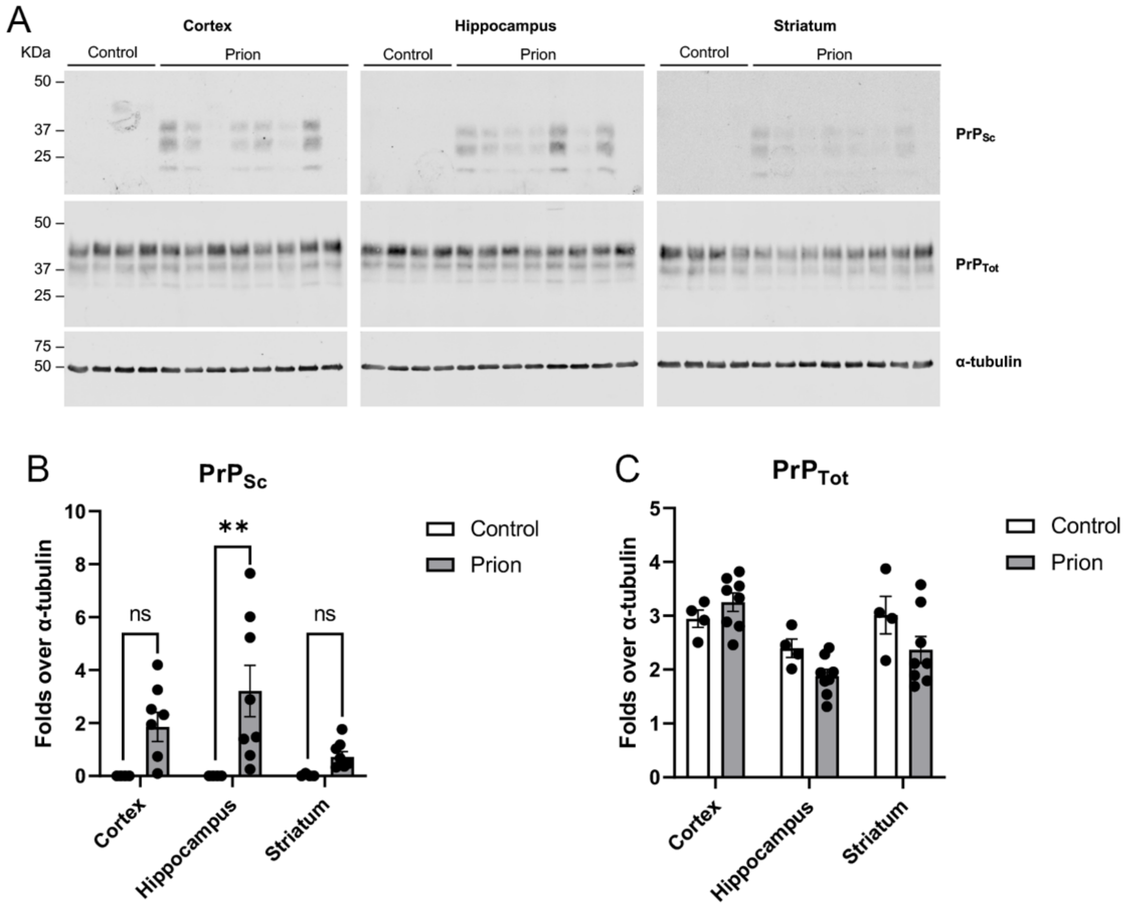

Figure S1: Prion-diseased mice display accumulation of scrapie prion 7 weeks after inoculation. (A to C) Lysates from cortex, hippocampus and striatum of control or prion-infected mice 7 weeks post inoculation (w.p.i.) were incubated in the presence or absence of proteinase K prior to Western blotting to detect non-digested scrapie prion protein (PrP_{Sc}) and total prion protein (PrP_{Tot}), respectively (A). Band intensity for PrP_{sc} (B) and PrP_{Tot} (C) quantified as fold change over band intensity of α-tubulin, shown as means ± SEM from n=4 to 8 mice; individual values are displayed within bars. **P<0.01 by two-way ANOVA Sidak Multiple comparisons.

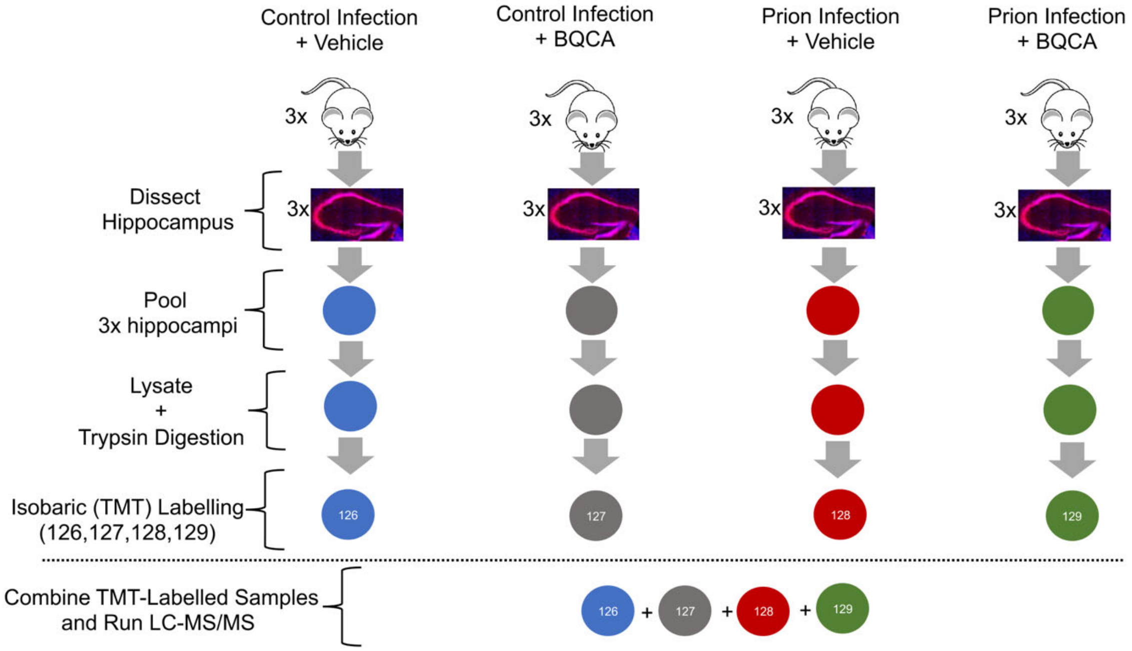

Figure S2: Proteomics schematic for BQCA experiments. Illustrated summary of the experimental outline and sample preparation for mass spectrometry-based proteomics analysis of hippocampi from vehicle and BQCA treated mice. Hippocampi from three mice in each of the experimental groups (control + vehicle, control + BQCA, prion + vehicle, and prion + BQCA) were pooled together, processed, and labelled with respective TMT, then combined and analyzed by LC-MS/MS to give one experimental run.

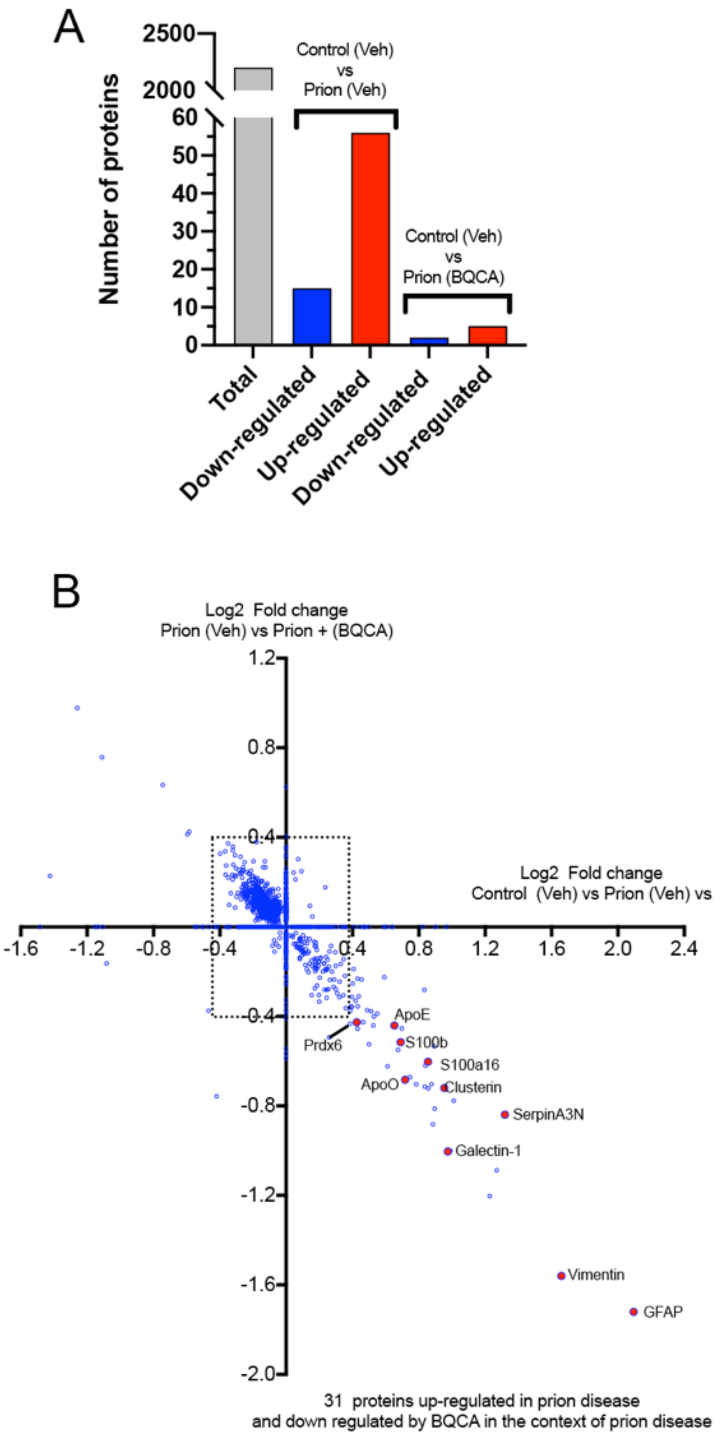

Figure S3: Different M$_1$-PAMs mediate similar effects in prion disease. (A) Number of proteins that are up- or down regulated in the “prion-effect” of vehicle- and BQCA-treated mice. **(B)** Quadrant scatter plot showing the effect of BQCA in the context of prion. The x-axis shows fold changes of protein expression that are up- or down-regulated by prion disease (comparison between control-vehicle and prion-vehicle; “prion-effect”); the y-axis shows fold changes of protein expression that are up- or down-regulated by BQCA in prion disease (comparison between prion-vehicle and prion-BQCA; “PAM-effect”). Proteins outside the square box are significantly changed in expression (FDR<0.05, ±Log$_2$ 0.4-fold change).

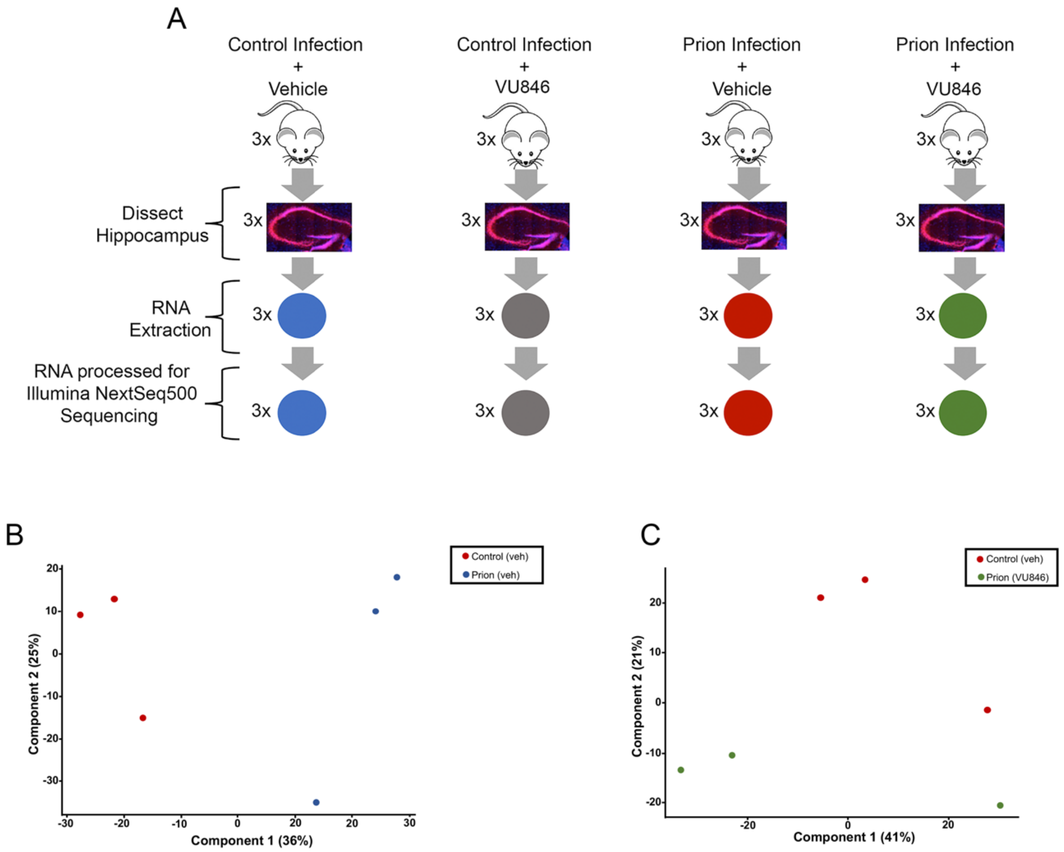

Figure S4: Transcriptomics schematic and PCA. (**A**) Illustrated summary of the experimental outline and sample preparation for the transcriptomics study. Hippocampi from three mice from each of four experimental groups [control + vehicle (Veh), control + VU846, prion + vehicle, and prion + VU846] were dissected and the RNA extracted and processed for Illumina NextSeq500 sequencing. (**B and C**) Principal components analysis (PCA) of the transcriptomics data from (B) 3 control + vehicle and 3 prion-infected + vehicle, and (C) 3 control + vehicle and 3 prion-infected + VU846.

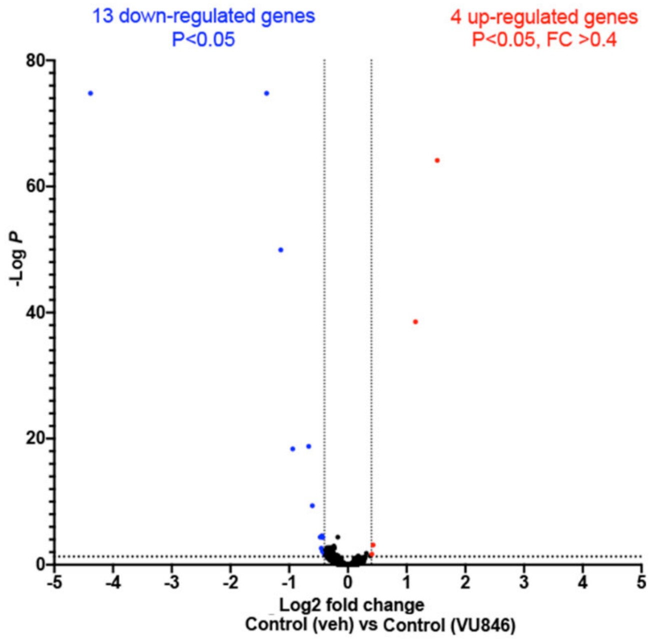

Figure S5: Transcriptomic "PAM-effect" in the control (non-infected) mice. Volcano plot generated using DESeq2 differential gene expression analysis of M_1-PAM in the context of control (non-infected mice). Red and blue represent genes with significantly increased and decreased expression, respectively, in control-vehicle versus control-VU846 (FDR<0.05, ±Log_2 0.4-fold change).

Antibody	***Source***	***Dilution***	***Company***	***Catalog No.***
GFAP	Mouse	1:5000	Sigma	G3893
Vimentin	Mouse	1:2000	R&D Systems	MAB21052
APOE	Rabbit	1:3000	Abcam	ab183596
SERPINA3N	Mouse	1:2000	R&D Systems	AF4709
Galectin-1	Rabbit	1:1000	Sigma	SAB5500110
Clusterin	Mouse	1:2000	R&D Systems	AF2747
Prion	Mouse	1:2000	Abcam	ab61409
α-Tubulin	Mouse	1:5000	Abcam	ab7291

Table S1. Primary antibodies used in Western blotting.

Data file S1: Proteomic analysis of hippocampi from prion-disease mice. Proteomics analysis showing the **(sheet 1)** total number of proteins quantified, **(sheet 2)** the proteins that are significantly changed by more than 0.4 log2 fold, **(sheet 3)** the Gene Ontology analysis of the significantly up-regulated proteins, and the Pathway Studio analyses for the **(sheet 4)** cell processes and **(sheet 5)** disease of the significantly up-regulated proteins in hippocampi from prion-disease mice. File is in .xls format in the online supplementary materials.

Data file S2: The effect of VU0486846 and BQCA on the proteome of hippocampi from prion-diseased mice. Proteomics analysis showing **(sheet 1)** the proteins that were significantly up-regulated by more than 0.4 log2 fold in hippocampi from prion+VU846 mice relative to control+vehicle mice, and **(sheet 2)** the proteins that were significantly different between prion-effect vehicle vs prion-effect VU846. Gene Ontology results of **(sheet 3)** the proteins that were significantly up-regulated in the context of prion disease but reduced or "normalized" by VU846 treatment, and **(sheet 4)** of proteins that were significantly down-regulated in the context of prion disease but upregulated or "normalized" by VU846 treatment. List of proteins that were significantly changed in **(sheet 5)** prion+VU846 vs prion+vehicle ("PAM effect") and those in **(sheet 6)** control+VU846 vs control+vehicle. List and analyses of **(sheet 7)** proteins that were up-regulated in hippocampi of mice with prion disease ("prion effect") and those that were down-regulated by VU846 ("PAM effect"). Lists and analyses of **(sheet 8)** all 2,202 proteins that met the criteria for quantification in the BQCA proteomics study, with those that were significantly up-regulated **(sheet 9)** or down-regulated **(sheet 10)** in prion disease. List and analysis **(sheet 11)** of all proteins that were significantly changed by more than 0.4 log2 fold in the prion+BQCA vs the prion+vehicle condition (PAM effect). Lastly, **(sheet 12)** a list of all proteins that were significantly changed by both BQCA and VU846 in the prion+vehicle vs prion+BQCA/VU846 conditions. File is in .xls format in the online supplementary materials.

Data file S3: Transcriptomics analysis of hippocampi from prion-disease mice. (Sheet 1) List of all 24062 genes that met the criteria for quantification. List of genes that are significantly **(sheet 2)** up-regulated or **(sheet 3)** down-regulated in prion disease. List of genes that are significantly changed in **(sheet 4)** prion-VU846 vs control-vehicle, and **(sheet 5)** control-VU846 vs control-vehicle. **(sheet 6)** List of all genes that are significantly different between prion-effect vehicle vs prion-effect VU846. Gene Ontology results (biological processes) of genes that are **(sheet 7)** significantly up-regulated in context of prion disease, but reduced or 'normalised' by VU846, or **(sheet 8)** significantly down-regulated in context of prion disease, but upregulated or 'normalised' by VU846 treatment. **(sheet 9)** Genes that are up-regulated in prion disease (prion-effect) and are down-regulated by VU846 (PAM-effect). File is in .xls format in the online supplementary materials.

Data file S4: Analysis of overlap between proteomics and transcriptomics datasets. Protein changes that unique to the proteomic data set and do not overlap with the transcriptomic data in the context of **(sheet 1)** prion effect, and **(sheet 2)** PAM effect. Transcriptional changes that are unique to the transcription data set and do not overlap with the proteomics datasets in the context of **(sheet 3)** prion effect, and **(sheet 4)** PAM effect. File is in .xls format in the online supplementary materials.
